# Where Is Your Attention? Assessing Individual Instances of Covert Attentional Orienting in Response to Gaze and Arrow Cues

**DOI:** 10.3390/vision1030019

**Published:** 2017-07-06

**Authors:** Christopher D. Blair, Francesca Capozzi, Jelena Ristic

**Affiliations:** Department of Psychology, McGill University, Stewart Biological Sciences Building, 1205 Dr. Penfield Avenue, Montreal, QC H3A 1B1, Canada

**Keywords:** attention, gaze following, eye direction

## Abstract

Humans spontaneously follow where others are looking. However, recent investigations suggest such gaze-following behavior during natural interactions occurs relatively infrequently, only in about a third of available instances. Here we investigated if a similar frequency of orienting is also found in laboratory tasks that measure covert attentional orienting using manual responses. To do so, in two experiments, we analyzed responses from a classic gaze cuing task, with arrow cues serving as control stimuli. We reasoned that the proportions of attentional benefits and costs, defined as responses falling outside of 1 standard deviation of the average performance for the neutral condition, would provide a good approximation of individual instances of attentional shifts. We found that although benefits and costs occurred in less than half of trials, benefits emerged on a greater proportion of validly cued relative to invalidly cued trials. This pattern of data held across two different measures of neutral performance, as assessed by Experiments 1 and 2, as well as across the two cue types. These results suggest that similarly to gaze-following in naturalistic settings, covert orienting within the cuing task also appears to occur relatively infrequently.

## 1. Introduction

Eye direction can convey a range of useful information—the location of a threat, interest of a mate, or a source of food [1]. As such, it is not surprising that humans follow where others are looking and orient their attention to gazed-at locations [1,2,3]. In the laboratory, gaze following is often measured using a computerized task, in which participants are presented with multiple trials featuring a central face cue looking in a particular direction (e.g., to the left or right) and are asked to respond to targets appearing at the gazed-at or not-gazed-at locations (e.g., [4]). A large number of studies conducted using this so-called ‘gaze cuing’ procedure show robust overall performance facilitation for gazed-at trials, as indexed by faster response times for targets appearing at gazed-at locations relative to those appearing at not-gazed-at locations, even when gaze direction remains fully task-irrelevant and does not convey any meaningful information about the target (e.g., [5,6]). This result has typically been interpreted as indicating that participants orient their attention in response to gaze cues consistently and spontaneously (e.g., [4]).

In contrast, observational studies suggest that gaze-following behavior during real world interactions does not appear to occur on every instance in which a deviated gaze cue is available. Recent research that has measured gaze following during natural behaviors shows that despite multiple opportunities, humans follow gaze cues in only a small fraction of instances (e.g., [7,8]). For example, Hayward et al. [8] measured the proportion of gaze following during a two-minute face-to-face conversation, and found that participants followed the directional gaze cues displayed by the conversational partner in about 30% of opportunities, which is consistent with estimates of gaze following reported by similar prior work (see [7,9]).

While this relative infrequency of gaze following has been theorized to reflect the modulating influence of contextual and social factors available during complex real world interactions (e.g., [7,10,11]), it is also possible that a similar frequency of occurrence may be observed during laboratory investigations as well. However, examinations of individual instances of attentional orienting during laboratory tasks have not yet been performed due to researchers typically analyzing participants’ average performance across all gazed-at and not-gazed-at trials (e.g., [2,4,5,12,13]). This stands in contrast to naturalistic procedures in which researchers analyze the proportion of discrete gaze following behaviors displayed by the participant relative to all available opportunities. As such, naturalistic studies reflect a proportion of gaze following behavior relative to all gaze-cue instances while laboratory data reflect average performance across all gazed-at and not-gazed-at trials. At present, the typical analysis of the laboratory data does not allow one to establish whether the overall gaze cuing effect reflects consistent gaze orienting throughout the task. It also does not yield an understanding of whether this overall orienting effect reflects a pattern in which attention is consistently oriented toward the target on the majority of gazed-at trials and away from the target on the majority of not-gazed-at trials.

To address this issue, in the present investigation we examined manual performance from a typical gaze-cuing task by analyzing the proportions of benefits and costs. We reasoned that the proportion of responses with reaction times falling outside of 1 standard deviation (SD) of the performance yielded by corresponding neutral trials (i.e., benefits and costs) would represent a good approximation of the frequency of individual indices of attentional orienting behaviors [4,14,15]. This follows from existing work, which shows that when attention is sufficiently biased, both benefits and costs emerge in manual performance with corresponding sensory gains found in early neural processing of the targets (e.g., [16]). As such, this provides a way to index the frequency of individual instances of attentional orienting during the typical cuing task by assessing the proportion of trials in which the attentional cue elicited a sufficiently large change in performance to be considered an attentional benefit or cost. It is worth noting here that this examination of responses does not represent a novel statistical procedure, but rather an additional way of summarizing and analyzing response time (RT) data. As such, all conventions governing the analyses employed on average RT data and those that we perform here on proportions of benefits and costs apply equally for both dependent measures.

**Figure 1:**
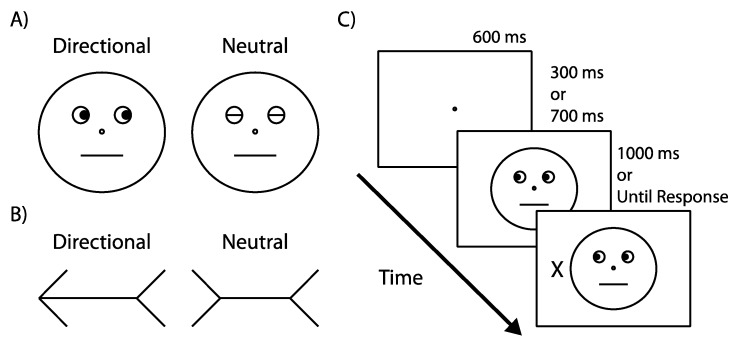
(**A**) Gaze cues; (**B**) Arrow cues; (**C**) Example trial sequence, illustrating a valid trial. All trials started with a 600 ms fixation display. Then a central cue, either a face or an arrow, was presented. After 300 or 700 ms, a response target, a capital letter ‘X’ or ‘O’, was shown at either the left or right peripheral location. Both the cue and the target remained on the screen until response or until the trial had timed out. Drawings are not to scale.

Participants were asked to complete a typical gaze-cuing task in which the central gaze cue indicated the correct target location randomly, on half of trials. To measure any potentially unique effects of this social cue, we also included an additional condition in which a nonsocial arrow served as a central cuing stimulus [5,17,18,19,20]. Figure 1 shows the stimuli and an example task sequence. In the gaze condition, a schematic face with left or right deviated gaze served as a central cue (Figure 1A). In the arrow condition, an arrow pointing left or right served as a central cue (Figure 1B). To measure benefits and costs, the neutral condition in Experiment 1 reflected performance in response to nondirectional cues (Figure 1A,B). In Experiment 2, the neutral condition reflected average performance in response to targets occurring along the not-cued dimension (i.e., up and down). Trials on which response time (RT) fell below 1 standard deviation (SD) relative to the average RT of corresponding neutral trials were labeled as benefits while those that fell above 1 SD were labeled as costs.

If the typical gaze-cuing effect reflects consistent orienting of attention throughout the task, we expected to observe benefits on at least half of valid trials and costs on at least half of invalid trials. We also expected to find benefits on a greater proportion of valid trials and costs on a greater proportion of invalid trials.

## 2. Results

### 2.1. Experiment 1

#### 2.1.1. Omnibus Analyses

Overall, participants performed the task well. Response errors, defined as anticipations (RTs < 100 ms), timed-out responses (RT > 2 SD of individual participant mean RT) and false alarms (responding on a no-target trial) accounted for 2.57%, 3.40%, and 8.56% of data respectively. Error rates did not differ between gaze (M = 14.40%, SD = 7.30%) and arrow cues (M = 14.64%, SD = 6.97%, *t*(24) = 0.13, *p* = 0.89, two-tailed, paired samples comparison). Errors were removed from the data and were not analyzed further.

**Figure 2:**
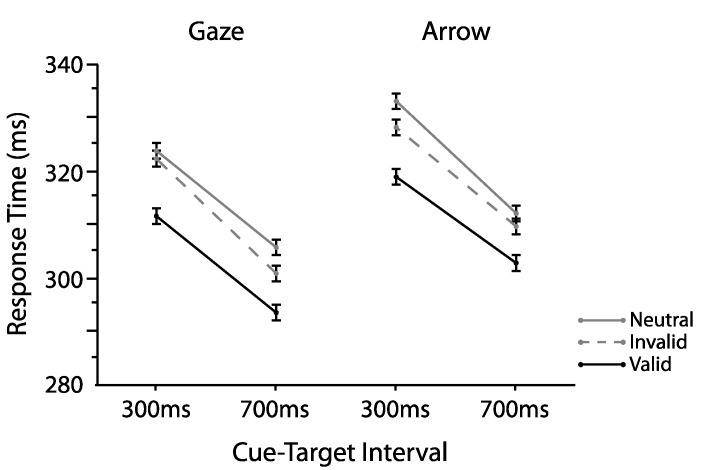
Mean response times (RTs) as a function of cue type, cue validity, and cue-target interval for Experiment 1. Error bars denote the standard error of the difference between the means.

We first validated our manipulation using omnibus analyses typically employed in similar studies [4,13,18,19,20]. To this aim, we examined interparticipant mean correct RTs as a function of cue type (gaze, arrow), cue validity (valid, invalid), and cue-target interval (300, 700 ms) using an omnibus repeated measures ANOVA. Figure 2 plots those means. As shown there, validly cued targets (M = 306.74, SD = 30.82) were detected faster relative to both invalidly cued (M = 315.09, SD = 34.20; *t*(24) = 4.66, *p* < 0.0001, two-tailed, paired, *d_z_* = 0.93) and neutral targets (M = 318.62, SD = 33.09; *t*(24) = 8.29, *p* < 0.0001, two-tailed, paired *d_z_* = 1.66; main effect cue validity *F*(2,48) = 27.47, *p* < 0.0001, η_p_^2^ = 0.53). A main effect of cue-target interval (*F*(1,24) = 40.12, *p* < 0.0001, η_p_^2^ = 0.63) indicated a typical foreperiod, with overall faster RTs for targets appearing at longer relative to shorter cue-target intervals (e.g., [21]). No other effects or interactions were reliable (all *Fs* < 3.93, *ps* > 0.59). Thus, both arrow and gaze cues facilitated target detection overall, in that validly cued targets were responded to faster compared to both invalidly cued and neutral targets. Furthermore, and replicating the large body of existing literature (e.g., [17]), no differences between orienting effects elicited by social gaze and nonsocial arrow cues were found (all interactions involving cue type; *Fs* < 1.22, *ps* > 0.30). Having demonstrated reliable differences between the mean RTs for valid and invalid trials, we next turned to investigations of the proportions of trials defined as benefits and costs.

#### 2.1.2. Proportion Analyses

**Figure 3:**
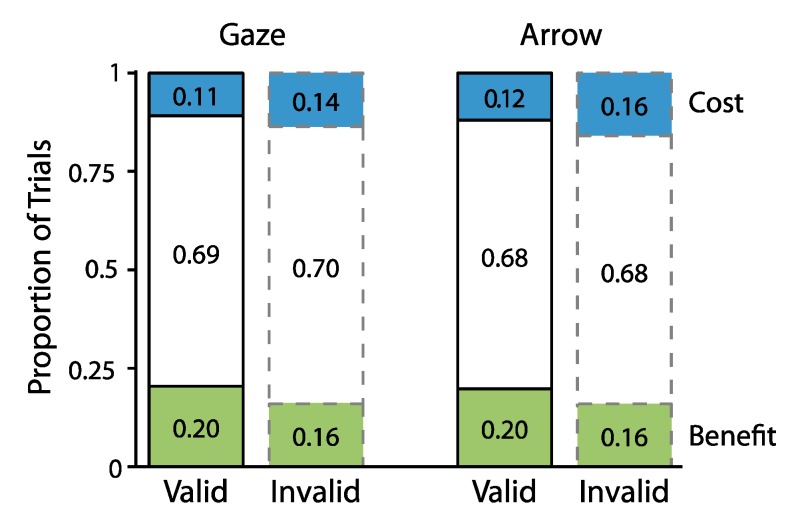
Cumulative proportion of trials for Experiment 1 classified as benefits (green), costs (blue), and the remaining trials (white).

To define benefits and costs for each participant, we first determined the proportion of valid and invalid trials for which individual participants’ RTs were slower and faster than the mean RT of the corresponding neutral trials (i.e., for each participant and individual condition). For example, RTs for valid and invalid trials from one participant in which the cue was an arrow, the cue-target interval was 300 ms, and the target appeared on the right were compared to the mean and SD of neutral trials for that same participant in which the cue was an arrow, the cue-target interval was 300 ms, and the target appeared on the right. Benefits were labeled as trials on which RT fell below 1 SD of the mean of those neutral trials. Costs were labeled as trials on which RT exceeded the mean of those neutral trials by at least 1 SD. Figure 3 depicts the cumulative proportion distributions of benefits, costs, and the remaining trials as a function of cue type and cue validity.

This breakdown enabled us to assess our hypotheses regarding the frequency of individual instances of attentional orienting. As outlined before, we expected to find benefits on at least half of valid trials and costs on at least half of invalid trials. To test this hypothesis, we compared the proportion of benefits (calculated as the number of valid or invalid trials falling below the 1 SD cutoff divided by the total number of trials of the same type (valid or invalid) for that participant) and costs (calculated as the number of valid or invalid trials exceeding the 1 SD cutoff divided by the number of trials of the same type (valid or invalid) for that participant) against the 50% mark using one-sample *t*-tests. We found that benefits occurred on 20% of valid trials (SD = 0.05) which was reliably lower than 50% (*t*(24) = 33.73, *p* < 0.0001, *d_z_* = 6.75). The same pattern held for costs. Costs occurred on 16% of invalid trials (SD = 0.05), which was also reliably lower than the 50% mark (*t*(24) = 72.87, *p* < 0.0001, *d_z_* = 14.58). Thus, both benefits and costs occurred on less than half of available valid and invalid trials, respectively.

**Figure 4:**
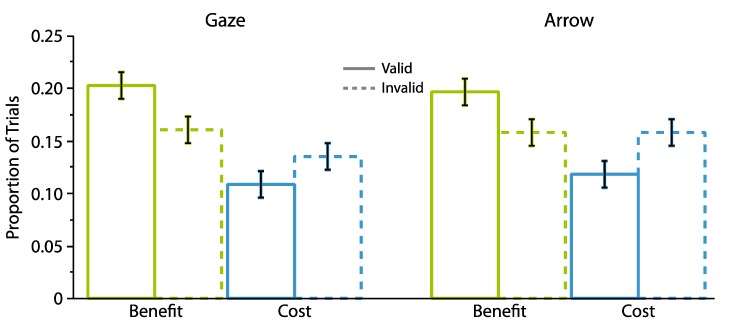
The proportion of benefits and costs as a function of cue type and cue validity for Experiment 1. Error bars represent standard error of the difference between the means.

To address our second hypothesis of whether a greater proportion of benefits occurred on valid trials and a greater proportion of costs occurred on invalid trials, we next analyzed how the proportions of benefits and costs varied with cue validity. To do so, we conducted a repeated measures ANOVA in which the proportions of benefits and costs were examined as a function of cue type (gaze, arrow), cue validity (valid, invalid), and cue-target interval (300, 700 ms). Figure 4 plots the results from this analysis. The data indicated an overall greater proportion of benefits relative to costs (Benefits: M = 0.18, SD = 0.04; Costs: M = 0.13, SD = 0.04; *F*(1,24) = 19.41, *p* = 0.0002, η_p_^2^ = 0.45). They also showed that both benefits and costs varied reliably with cue validity (*attentional orienting x cue validity F*(1,24) = 18.10, *p* = 0.0003, η_p_^2^ = 0.43), in that a greater proportion of valid relative to invalid trials (0.20 vs. 0.16) were classified as benefits (*t*(24) = 3.69, *p* = 0.001, two-tailed, paired, *d_z_* = 0.74), while a greater proportion of invalid relative to valid trials (0.15 vs. 0.11) were classified as costs (*t*(24) = 3.93, *p* = 0.0006, two-tailed, paired, *d_z_* = 0.79). An absence of interactions with cue type suggested that the same pattern of data held across gaze and arrow cues (*cue type x attentional orienting x cue validity*, *F*(1,24) = 0.09, *p* = 0.77, η_p_^2^ = 0.004). No other effects were significant (0.13 < *p* < 0.93).

Together, the results from these analyses show that benefits and costs occurred relatively rarely. However, more valid trials relative to invalid trials were classified as benefits and more invalid trials relative to valid trials were classified as costs. This is consistent with an observation that the overall RT performance facilitation for valid targets typically found in the cuing task likely reflects participants initiating attentional orienting sporadically rather than consistently throughout the task. In Experiment 2, we assessed if our choice of the neutral condition influenced this result.

### 2.2. Experiment 2

While the approach we employed in Experiment 1 followed the standard procedures for eliciting and measuring attentional benefits and costs, this analysis relied on comparisons between two physically different cues (i.e., directional vs. non-directional; e.g., [14]). As such, it is possible that RTs in the neutral case did not represent a reliable comparison for trials in valid and invalid conditions. One way to mitigate this concern would be to keep the physical cue constant across the comparisons and to contrast responses across multiple possible target locations (i.e., valid; invalid; not-cued; e.g., [13,22]). We employed this alternative in Experiment 2.

To do so, we utilized a four-location cuing task in which, like in Experiment 1, cue direction was manipulated across the horizontal axis but the targets could appear across both horizontal and vertical axes, i.e., left, right, top, and bottom positions. The targets appearing in the left or right locations were those that were associated with valid and invalid cues. The average response for the targets appearing in the top and bottom positions constituted a neutral comparison, as responses drawn from those trials followed the presentation of a physically identical cue to those presented on valid and invalid trials. All other procedures and analyses mirrored Experiment 1.

#### 2.2.1. Omnibus Analyses

As before, response errors were infrequent (Anticipations, Gaze: 1.99%, Arrow: 2.34%; Timeouts, Gaze: 3.90%, Arrow: 3.82%; False alarms, Gaze: 5.17%, Arrow: 4.17%) and did not differ across cue conditions (*t*(24) = 0.92, *p* = 0.37).

**Figure 5:**
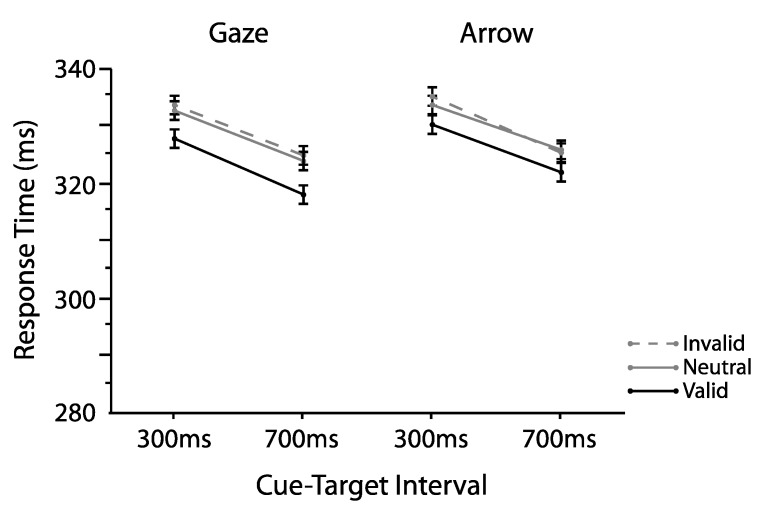
Mean RTs as a function of cue type, cue validity, and cue-target interval for Experiment 2. Error bars denote the standard error of the difference between the means.

Figure 5 shows mean correct interparticipant RTs as a function of cue type, cue validity, and cue-target interval. The omnibus repeated measures ANOVA indicated a main effect of cue validity (*F*(2,48) = 10.00, *p* = 0.0002, η_p_^2^ = 0.29), with responses on validly cued trials (M = 324.40, SD = 44.30) overall reliably faster than responses on both invalidly cued (M = 329.38, SD = 46.24, *t*(24) = 3.33, *p* = 0.003, two-tailed, paired *d_z_* = 0.67) and neutral trials (M = 328.82, SD = 44.87, *t*(24) = 3.35, *p* = 0.003, two-tailed, paired, *d_z_* = 0.67). The main effect of cue-target interval was reliable once again (*F*(1,24) = 14.10, *p* = 0.001, η_p_^2^ = 0.37) with no other effects or interactions (all *Fs* < 0.52, ps > 0.60). Thus, as in Experiment 1, both gaze and arrow cues produced reliable and equivalent orienting effects overall (all interactions involving cue type, *Fs* < 0.52, *ps* > 0.60).

#### 2.2.2. Proportion Analyses

**Figure 6:**
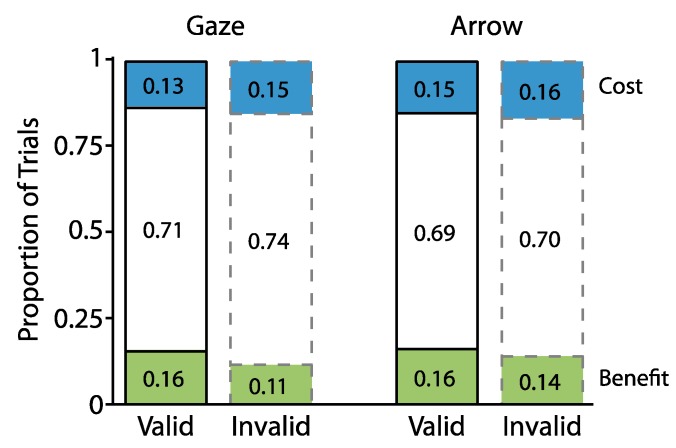
Cumulative proportion of trials for Experiment 2 classified as benefits (green), costs (blue), and other trials (white).

To examine the proportions of benefits and costs, the data were subjected to the same procedures as in Experiment 1. The results fully replicated Experiment 1. First, and as illustrated in Figure 6, which shows cumulative proportions of trials classified as benefits and costs, once again we found that benefits occurred on less the 50% of valid trials (M = 0.16, SD = 0.05, *t*(24) = 35.98, *p* < 0.0001, two-tailed, one sample *d_z_* = 7.20) and that costs occurred on less than 50% of invalid trials (M = 0.16, SD = 0.03, *t*(24) = 53.77, *p* < 0.0001, two-tailed, one sample, *d_z_* = 10.75).

**Figure 7:**
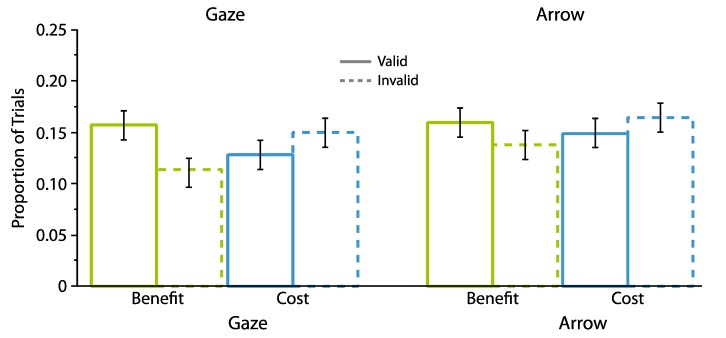
The proportion of benefits and costs as a function of cue type and cue validity for Experiment 2. Error bars represent standard error of the difference between the means.

Second, a repeated measures ANOVA, which examined the proportions of benefits and costs as a function of cue type (gaze, arrow), cue validity (valid, invalid), and cue-target interval (300, 700 ms) returned a main effect of cue type (*F*(1,24) = 9.55, *p* = 0.005, η_p_^2^ = 0.28) and a reliable interaction between attentional orienting and cue validity (*F*(1,24) = 16.99, *p* = 0.0004, η_p_^2^ = 0.42). As illustrated in Figure 7, this interaction indicated that a greater proportion of valid (M = 0.16, SD = 0.05) compared to invalid trials (M = 0.13, SD = 0.04) were classified as benefits (*t*(24) = 4.41, *p* < 0.0001, two-tailed, paired, *d_z_* = 0.88), while a greater proportion of invalid (M = 0.16, SD = 0.03) relative to valid trials (M= 0.14, SD = 0.04) were classified as costs (*t*(24) = 2.55, *p* = 0.02, two-tailed, paired, *d_z_* = 0.51). No other effects were reliable (all *Fs* < 3.05, 0.09 < *p* < 0.90).

Thus, as in Experiment 1, results from Experiment 2 indicated that benefits and costs occurred on fewer than half of available trials, that more valid relative to invalid trials were classified as benefits, and that more invalid relative to valid trials were classified as costs. As such, this result supports the data from Experiment 1 and indicates that this main finding held similarly across two different neutral condition comparisons.

## 3. Discussion

In this study, we sought to index individual instances of covert attentional orienting within the gaze-cuing task. To this end, we examined participants’ manual performance as a function of benefits and costs, defined as participants’ responses falling below and above 1 SD of their average neutral RT. Based on past research [4,14,15,16] we reasoned that trials labeled as benefits and costs would provide a reasonable estimate of individual instances of attentional orienting, as on those trials, attention was biased strongly enough by the cue to generate a facilitative or detrimental effect on performance. Across two different neutral conditions, manipulated in Experiments 1 and 2, we found that both benefits and costs occurred on fewer than half of valid and invalid trials respectively. Furthermore, we also found that benefits occurred more frequently on valid vs. invalid trials, and costs occurred more frequently on invalid vs. valid trials. Together, these findings suggest three general implications.

One, our result indicating that benefits and costs occurred on fewer than half of available trials suggests that the typical average orienting effect yielded by the cuing task does not reflect consistent attentional orienting throughout the task, and dovetails well with the results from observational studies [7,8]. In other words, it appears that the measure of average orienting yielded by the cuing task may be driven by instances of attentional orienting occurring on a minority rather than a majority of trials. It is possible, however, that this result reflects our conservative estimate of the individual instances of attentional orienting, as we defined benefits and costs as responses that deviated from the average performance in a relatively extreme fashion (by 1 SD). To probe into this issue, we analyzed the data using a more liberal benefit and cost comparison cutoff of 0.5 SD. Not surprisingly, applying this lower benchmark resulted in overall increased proportions of benefits and costs, which together now constituted 61.68% and 61.30% of trials in Experiments 1 and 2, respectively. However, despite this overall increase, the proportions of benefits and costs still mirrored the main results. Specifically, while benefits now occurred on 42.03% of valid trials in Experiment 1, and on 38.13% of valid trials in Experiment 2, both of these values were still statistically lower than the 50% mark (E1: *t*(24) = 7.62, *p* < 0.0001, *d_z_* = 1.52; E2: *t*(24) = 10.65, *p* < 0.0001, two-tailed, one sample tests, *d_z_* = 2.13). The same held for costs, which occurred on 25.85% and 26.47% of invalid trials in Experiments 1 and 2, respectively. Again, both of these values were still reliably lower than 50% (E1: *t*(24) = 17.56, *p* < 0.0001, *d_z_* = 3.51; E2: *t*(24) = 28.18, *p* < 0.0001, *d_z_* = 5.64). Thus, even when the criterion for identifying benefits and costs was lowered using a more liberal 0.5 SD cutoff, benefits and costs still did not occur on a majority of trials. As such, this provides support for the notion that the average orienting effect yielded by the cuing task likely does not reflect consistent attentional orienting throughout the task, but isolated incidences of strong performance biases occurring on less than half of available trials.

Second, although we found that benefits and costs occurred on a minority of trials, their representation across valid and invalid trials was consistent with a pattern predicted by the available literature. Namely, our data indicated that more benefits relative to costs occurred on valid trials and that more costs relative to benefits occurred on invalid trials. This supports our initial hypothesis and validates the present analytical approach. More specifically, the results of our proportion analyses dovetail with the conclusions from typical analyses of average performance while providing a more detailed characterization of the dynamics of attentional orienting behaviors. As such, this method may be useful for future studies investigating which characteristics of attentional cues may modulate the frequency and/or strength of instances of covert attentional orienting.

Finally, like many past studies [18,20,23], our examination also did not reveal reliable differences across gaze and arrow cues. Unlike orienting to gaze direction, which is thought to reflect evolutionarily driven social biases [1,2,3,5], attentional orienting in response to arrows is purported to reflect an automated process, which arises as a function of overlearning the meaning of behaviorally relevant symbols [24,25,26]. Despite this fundamental difference, cuing tasks continue to show similar overall performance across gaze and arrow cues ([18,20,23]; but see [19]). The present investigation is the first to our knowledge to examine whether the two types of cues may also differ in individual instances of orienting. Our results for both gaze cues and arrow cues were once again indistinguishable. They indicated the presence of benefits and costs on less than half of valid and invalid trials, with the proportion of benefits on valid trials ranging between 16% and 20%, and the proportion of costs on invalid trials ranging between 14% and 16% for both gaze and arrow cues across the two experiments. Additionally, when we compared the proportions of benefits and costs across valid and invalid trials, the absence of significant interactions involving cue type for each experiment indicated that the result showing a greater proportion of benefits relative to costs on valid trials and a greater proportion of costs relative to benefits on invalid trials was not preferentially driven by the social or automated cue type.

It is nevertheless important to interpret these results within the context of two broad methodological considerations. First, we remain mindful of the fundamental differences between real-world and laboratory investigations due to naturalistic investigations measuring overt orienting directly and laboratory performance investigations measuring covert orienting indirectly. As such, the contribution of covert orienting in the overt effects assessed during naturalistic studies remains relatively unknown (e.g., [7,8]). In turn, it is possible that the frequency of gaze following occurrence in naturalistic settings may be underestimated, a point that will be important to address in future research (see [27,28] for discussion). Systematic comparisons between laboratory and naturalistic studies in which orienting in response to discrete instances of attentional cues is manipulated and measured using both covert and overt measures are needed to establish the correspondence across these approaches (see [8] for a recent study on this topic). Second, one also needs to remain mindful of the potential role of task parameters. Here, we employed a set of task parameters which have been used by many past gaze- and arrow-cuing studies [4,12,13,18,19,20]. While this allows for establishing meaningful links between the present data and the existing literature, it also raises a question of whether alterations of these typical parameters may lead to changes in the estimated prevalence of attentional orienting. Increasing task difficulty, changing the nature of the response task (discrimination vs. detection), and/or increasing the realism of the stimuli (photographs vs. schematic faces) are some of the potential factors that would be worth examining.

An intriguing outstanding question posed by this work relates to the notion of *why* attentional orienting does not appear to be elicited by each available cue instance. This is particularly puzzling within the context of the traditional attentional theory, in which a reflexive or spontaneous orienting response is purported to reflect an automatic alignment of the orienting mechanisms with biasing occurring within the corresponding neural pathways (e.g., [29]). In the case of social attention, it has now been consistently demonstrated that such social attentional effects do not occur obligatorily but rather in a contextually and situationally appropriate manner [8,30,31,32]. It would be interesting to examine if similar patterns of sporadic attentional performance biasing are also observed when other attentional cues such as luminance increments are used. However, in both cases, the current methodology enables the examination of those performance patterns and permits future investigations of the factors that may influence and determine when an attentional shift is elicited by the cue. Such performance effects could also be studied using neuroimaging approaches, which may afford further opportunities to distinguish between the neural signatures of attentional effects using single-trial analyses [33,34,35]. In turn, understanding the underlying mechanisms driving performance changes in laboratory attentional paradigms will facilitate the creation of a much-needed methodological bridge between naturalistic and laboratory approaches (e.g., [8]) and will allow for a fine-grained characterization and measurement of attentional behavior in both the real world and the laboratory (e.g., [36]).

## 4. Materials and Methods

### 4.1. Experiment 1

#### 4.1.1. Participants

Twenty-five observers (1 Male, Mean age = 20.56, SD = 1.26) took part in the 1-h experiment in exchange for course credit. All procedures were in accordance with the Declaration of Helsinki (2008) and were approved by the McGill University Behavioral Research Ethics Board (#81-0909).

#### 4.1.2. Apparatus & Stimuli

All stimuli were black line drawings shown against a white background. Stimulus presentation and data collection were controlled by MATLAB’s Psychophysics Toolbox [37]. Stimuli were presented on a 16-inch cathode ray tube (CRT) display at an approximate viewing distance of 57 cm.

The face consisted of a circular outline subtending 6° × 5.6°, with two smaller outlines representing the eyes (0.79°), a central circle representing the nose (0.18°), and a horizontal line representing the mouth (1.94°). Black filled-in circles (0.44°) positioned either at the right or left outer edge of the eye circles represented gazing pupils. In the neutral condition, horizontal lines extending across the diameter of the eye circles represented closed eyes. The arrow (4.5°) consisted of a straight line (3.5°) with an arrowhead and arrow tail (1°). In the neutral condition, a straight line (2.5°) was centered between two inward pointing arrow tails (1°). Capital letters “X” and “O” (1° × 0.75°) served as response targets. They were positioned peripherally 6° to the left or right of fixation along the horizontal axis, as measured from the center of the screen to the center of the target.

#### 4.1.3. Design

The study was a repeated measures design with cue type (gaze, arrow), cue validity (valid, invalid, neutral), cue-target interval (300 and 700 ms), and target type (X, O) manipulated as factors. Cue type was blocked, with the order of presentation counterbalanced between participants. Cue validity, cue-target interval, and target type were intermixed and presented equally often within each cue condition. Cues could indicate either a left or right peripheral location. The target could appear at either the left or right peripheral location. Cue validity reflected the match between the cue direction (left; right; neutral) and target location (left; right). Trials on which the target occurred at the location indicated by the gaze/arrow cue were labeled as valid; those on which the target occurred at the location not indicated by the gaze/arrow cue were labeled as invalid; finally, trials on which targets occurred in either the left or right locations following the neutral cue were labeled as neutral. Cue-target interval reflected the time between the presentation of the cue and the presentation of the target. Following from past studies (e.g., [4,17]), we included one short (300 ms) and one longer cue-target interval (700 ms) to capture attentional effects of gaze and arrow cues, both of which have been found to be robust at each of these cue-target delays.

Cue type, cue direction, target position, cue-target interval, and target type were manipulated equally and equiprobably. As such, both gaze and arrow cues were fully spatially uninformative as to the target location and its identity, and participants were fully informed about this prior to completing the cuing tasks.

#### 4.1.4. Procedure

Figure 1C illustrates a typical trial. First, a 600-ms fixation display was shown. Then a central cue, either a schematic face or an arrow, appeared. After 300 or 700 ms, a response target was presented in either the left or right peripheral location. The cue and the target remained visible until participants responded or 1000 ms had elapsed. The intertrial interval was a random duration between 750 and 1000 ms.

Participants were instructed to maintain central fixation and to press the spacebar on the keyboard as quickly and accurately as possible once they detected the onset of the target. No target was presented on about 6% of trials, and participants were instructed to withhold a response in these instances. A total of 306 trials were run for each cue type, divided equally over three testing blocks. Fifteen practice trials were run at the start of each cue type block. RT was measured from target onset.

### 4.2. Experiment 2

#### Participants, Apparatus, Stimuli, Design, Procedure

Twenty-five new volunteers (2 Males, Mean age = 20, SD = 1.08) completed the study. All procedures were identical to those used in Experiment 1 except that: (i) the neutral cue was not used; (ii) the targets could appear above, below, to the left, and to the right of fixation; and (iii) to account for the increase in the number of target locations, participants completed 432 trials in response to each cue, divided equally over four testing blocks.

## 5. Conclusions

In this study, we presented a way for assessing individual instances of covert attentional orienting. Our data replicated typical overall patterns and additionally indicated, as theoretically predicted, that performance benefits occurred more frequently on validly cued trials while performance costs occurred more frequently on invalidly cued trials. However, the data also showed that these instances of attentional orienting did not occur on a majority of trials, but rather sporadically in less than half of available instances. This approach bodes well for future studies looking to understand the dynamics of attentional effects within and across real world and laboratory investigations.
